# Clinic of Jefferson Medical College

**Published:** 1851-08

**Authors:** G. R. Morehouse


### CLINICAL REPORTS.

*Clinic of Jefferson Medical College.*—Service of Profs. MÜTTER and PANCOAST. Reported by G. R. Morehouse, M.D.

### *Clinic of February* 15, 1851.

Cases presented this day:—Luscitas; oscillation of the eyeball; ophthalmia neonatorum; tendinous paronychia; caries of the tibia; two cases of strabismus, (convergens and divergens); hæmorrhoids; chronic inflammation of ligaments of the ankle; cystic goitre; extensive laceration of the hand; osteo-sarcoma of fibula; aneurism by anastomosis; epulis; varicocele; cataract; tertiary syphilis; abscess under the biceps flexor cubiti; glaucoma; caries of of the vomer; granular conjunctivitis; fungus hæmatodes of the eye; encanthis; necrosis of the femur; erysipelas; onyx; functional derangement of the cerebellum; caries of spine; varicose veins of leg; amaurosis. Total 30.

The following cases, after the clinical remarks as given below, were operated upon before the class by Professor Pancoast.

*Case* 1*st. Cystic Goitre.*—John Flinn, aet. 42, originally from the limestone region of this State, has, for some time past, been troubled with hypertrophy of the thyroid Gland. From the account he gives us, we gather this history of his case.

Before being subjected to any treatment, the tumor presented the usual appearance of goitre—a soft, painless, elastic swelling, occupying the position of the thyroid gland, of moderate bulk, and devoid of any signs of acute inflammation. Disliking the deformity, however, and dreading its further growth, the patient sought medical aid. He was placed for a long time under the internal and local use of iodine, which did not act so beneficially in this case as it is wont to do; the tumor seemed at first somewhat diminished, but it soon began to increase anew, and became firmer and harder. The skin covering the tumor became so red and tender under the use of the local application, that his physician deemed it advisable to discontinue the remedy. The burnt sponge was not used in this case, which I believe even more efficacious than the iodine, nor was any local depletion made, which it seems to me was indicated by the unusual inflammatory character of the case, as evinced by the rapidly increasing redness and hardness.

As it presents itself now, the edges of the tumor are strongly defined, both lateral lobes are very prominent, hard, and firmly adherent to the skin, which is red, shining, and extremely sensitive to the touch. In the isthmus, which is nearly as broad as the gland, there is a round fluctuating tumor, near the top of the sternum, about the diameter of a dollar, evidently a cyst developed in the tissue of the gland. The patient suffers greatly, respiration is seriously interfered with by the pressure of the enlarged and hardened mass upon the trachea, the return of the blood from the head by the veins is impeded from the same cause, producing a fulness of the vessels of the face and neck, and giving the patient a dark, puffy countenance, as of a man half-strangled. I look upon this case, as one of the most serious character. The excessive hardness of the mass, and the close adherence to it of the sensitive and violaceous skin, unusual circumstances in goitre, lead me to fear the disease is of malignant character, and to form an unfavorable prognosis—for cancer of the thyroid gland, though very rare, is sometimes met with. What can we do for the patient? The extirpation of the gland entire, a thing, many times attempted, sometimes done, is not to be thought of. It would be accompanied with such imminent risk to life, that no judicious surgeon, even in a case much more favorable than this, would attempt it.

To cut down and tie the superior thyroid arteries, an uncertain remedy at best, though not difficult in ordinary cases, is nearly impracticable here, from the size and unyielding character of the tumor. I believe, therefore, we must rely upon local depletion, and the use of an alterative pill internally, consisting of a half grain of the protiodide of mercury; two grains of gum guiac., and a third of a grain of opium, three times a day, keeping the patient quiet, and giving him a well regulated regimen. The cyst at the furca of the sternum, which is a very sensitive point, seems to press upon the trachea and must be otherwise disposed of. The cystic tumors of a goitre, require and justify some operative measure. Though they may have obtained considerable size, they have originally sprung from the cellular spaces of the gland that have undergone great enlargement, and may therefore be attacked with much more safety than a solid growth in the organ, which would be found more vascular. They may be treated with a seton, as we treat hydrocele of the scrotum and neck. This I have several times done with success, but it is attended with considerable risk, from the constitutional irritation it excites, and from the purulent absorption which may follow, both of which have occasioned death. They have been injected with iodine, but this is more hazardous still, and as erysipelas is very liable to follow, few surgeons would repeat the experiment. Prof. Porta, of Padua, in Italy, has proposed in a recent work, to dissect out these cysts. This, which at first view, would seem a very hazardous proceeding, from the very vascular character of this blood-gland, is really, a most judicious suggestion. For the cyst, you must recollect, springs from a small origin, and is really, rather a new development in, than a part of the proper structure of the gland.

I have recently put this suggestion in practice and succeeded in effecting, what I believe to be a final cure of a large goitre, in a young lady of this city. The enlargement in this case was principally confined to the right side of the gland, and was of several years standing. In the first instance, it was a solid tumor, accompanied with great anemia, a rapid fluttering pulse, and protrusion of the eye balls. By a varied course of medical treatment extending through eighteen months, I was enabled to overcome these latter symptoms, and greatly reduce the size of the gland. Then followed a cystic tumor, in the same position as the one in the case before us. This I finally obliterated by passing a seton through it, which I kept in for several months. Before the ribbon was withdrawn, another cyst was developed near the top part of the right thyroid lobe, which grew uninfluenced by any treatment I applied, till it reached the size of a pullet's egg. From its high position in the neck, the deformity was so conspicuous, that its removal in some manner was earnestly desired by the patient and her friends. I determined on reflection to attempt its extirpation after the plan of Professor Porta, for I deemed this, if cautiously done, not attended with greater risk than the use of the seton, which in the treatment of the former tumor had caused great constitutional irritation. If I could not remove the cyst entirely without provoking dangerous hæmorrhage, I determined, after laying it bare, to cut away a portion of its walls, and dress it from the bottom with lint. With the assistance of Dr. Ellerslie Wallace, I performed the operation in this way. I first made a free vertical cut through the skin, over the longitudinal axis of the tumor. I next cautiously divided the fasciæ and the platysma muscle, which was very red, thick and vascular, over a grooved director. The tumor thus exposed, presented a blue or livid appearance, from the coagulated blood with which it was filled within. I opened the cellular tissue over the top of the gland, and with a blunt strabismus hook, drew out and tied the superior thyroid artery, which was of large size. At the outer edge of the gland, and about its middle, I found a strong pulsating branch coming up from the inferior thyroid. This likewise I isolated and tied. I next proceeded to the separation of the cyst, grasping it with the forceps, and working it loose at its circumference from the body of the gland with the handle of the scalpel. I found its attachments very feeble, little or no stronger than those of the follicular tumors of the scalp, which you have so often seen Dr. Mütter and myself turn out in this arena. Under the grasp of the forceps the walls of the cyst gave way, and its contents were discharged. The sac collapsed a little, and I finished by detaching the tumor, otherwise entire, from its bed in the gland, with the finger nail. A little arterial bleeding followed, from the bottom of the deep hole left in the gland. By introducing two tenacula at right angles with each other, and throwing a ligature around them, this was at once suppressed. One or two other minute arterial twigs were tied in the substance of the platysma muscle. Very little blood was lost. The wound was lightly dressed; some suppuration followed, but in the course of three weeks, the cicatrization was complete. If no other tumours should follow, the patient will escape with only the marks of the incision, and a thyroid gland a little fuller than natural, for I believe when once greatly hypertrophied, this organ seldom, under any treatment, goes down to its normal size. If cysts should form subsequently in other parts of the gland, I would most certainly endeavor to remove them by the same process. This is, so far as I know, the only instance of a dissection of a cyst from the thyroid made in this country. But it has been repeatedly done by Professor Porta, and others in Italy, and the operation though requiring great caution, and knowledge of the structure on the part of the operator, is not difficult. In the case before us to-day, I would not however venture upon the operation. The prospect of a cure, is, I believe under any plan uncertain. I shall therefore merely lay open the cyst by an incision, and discharge its contents, so as to relieve the pressure on the trachea. I will then fill the cavity lightly with lint, and when the bleeding ceases poultice it, in order to promote suppuration and granulation. I treated successfully, about three years ago, a similar case in this place, after this manner, though I was then compelled to make repeated application of the vegetable caustic, to destroy fungous growths from the part. The cyst was opened, and its contents of coagulated blood, pus and serum evacuated. Its lining membrane was found studded with soft fungous excrescences, nearly filling its cavity. A dossel of lint was placed in the wound to check the bleeding, retained by a strip of adhesive plaster.*

*Case 2d. Paronychia.*—Rosanna Gillespie, æt. 39, presents herself with the worst form of whitlow, arising from a punctured wound of the finger. Cases of this sort are common occurrences,

*The result in this case seems likely to verify the unfavorable opinion of it above expressed. The patient experienced considerable immediate relief from the operation, as the tumor was much diminished and the breathing easier. But when last seen, some three months afterwards, a fungous growth of bad appearance had sprouted from the suppurating wound, for which a chloride of zinc paste was ordered.

and, therefore, more important to you, inasmuch as they will meet you at your first entrance into practice, and, if not properly managed, may terminate seriously, either by the loss of the finger, hand, or arm; or even of life itself, when the absorbents become extensively involved. In the present case the first stage has hardly passed; matter has formed beneath the vaginal ligaments and within the tendinous sheath of the finger, and has extended itself into the sero-cellular envelopes of the tendons in the palm. The constitutional disturbance has been very great, the glands in the axilla, and along the upper margin of the clavicle, are enlarged; the hand and wrist are greatly swollen; and here and there, along the arm, can be traced the red lines of commencing angeioleucitis.

Now, gentlemen, to understand this affection thoroughly, you should comprehend the *modus agendi* by which a trifling puncture lights up so severe a malady. The first effect of the puncture is inflammation with throbbing, which, if the puncture is not deep, is seated merely beneath the skin, but is exquisitely painful, from the number of nervous fibrils, pacinian bodies, and papillæ spread over the finger. The skin, from the thickness of the cuticle in this position, does not yield readily to distension, hence a lye poultice, by softening the cuticle, often tends, in the early stage, to assuage the pain. Sooner or later, if not arrested, the inflammation extends itself beneath the vaginal ligaments, involving the double synovial sheaths and trabecular threads of the tendons. The ligaments possessing no elasticity, do not yield to the increasing bulk of the inflamed tissue beneath; strangulation is therefore produced, and we have, as a consequence, sloughing of the tendinous sheaths, and of the periosteum lining the bottom of the vaginal grooves, with subsequent death of the phalanges. This constitutes a felon, in common language. But the disease, though compressed laterally, extends itself readily enough upwards into the palm, following in the sheath of one finger up into the common sero-cellular envelope of all the tendons of the hand and wrist. These parts swell enormously, and in this state are compressed by the bones on the one hand and the palmar fascia and the annular ligament on the other, producing agonizing suffering, and rendering tetanus liable, from strangulating pressure upon the nerves. The confined inflammation in the centre of the palm is now disposed to extend itself down the thecal bursæ of the other fingers and thumb, and along the tendons of the flexor muscles, high up the forearm; and in two instances, I have seen it spread over the bellies of the muscles, and finally destroy the ligaments of the elbow joint, and the interosseous ligament of the forearm. Under such circumstances the life of the patient is in great danger, and there is imminent risk of mortification of the hand and arm. Now, how are we to arrest all these terrible consequences, for, to some extent, they will surely follow in the case before us, if relief is not afforded.

The first, and most obvious, indication to be fulfilled, is the relief of the strangulation. This is to be done by entering the curved sharp-pointed bistoury, vertically through the skin, down through the vaginal ligaments, to the flexor tendons. Then bring the bistoury, with the back toward the palm, and let the point slide under the ligament, in the direction of the end of the finger, and then out through the skin. Let the incision extend the length of one phalanx; if it were made the whole length of the finger, the tendons would start out from the sheath. If more than one opening on the finger is required, it is best to make a separate puncture over another phalanx.

In this case, I make a free opening over the first phalanx of the second and third fingers, which particularly suffer. A considerable amount of pus escapes, as you see, and I am able to press more from the palm of the hand, rendering it clear to you that I have cut into the bursal sheaths of the tendons. Still the general purulent mass, which has collected in the palm, may not be sufficiently well discharged from these incisions, as we know the thecal bursæ are not open channels, but have here and there septal attachments, which connect them to the tendons. How, then, if this should follow, are we to free the palm? Lay it open as we have done the fingers? No! That would effectually relieve the strangulation and let out the matter, and we all know, as in this other case of hand, with extensive laceration, which I now show you, that a violent injury of the hand, as that made by the bursting of a gun, provided it opens the palmar fascia thoroughly, does, if judiciously treated, nearly always, well. But if we were to lay open the palm, by an incision, in the subject before us, we should inflict great pain, leave a deforming and troublesome cicatrix, which ought if possible to be avoided, and give rise to a troublesome hæmorrhage by the division of the ulnar arch, an arterial trunk which bleeds profusely when cut, but like other arteries bleeds little when violently torn. But when the middle palmar fascia becomes involved, as it sometimes does at one of its sides, so as to let a soft fluctuating tumor form in the palm, the tumor must then be opened, as the violence of the inflammation necessary to produce this result, will have effectually blocked the vessels of the part. I have a much better way, when the palmar fascia has not yielded, by which I have several times afforded complete relief in this state of the hand, without any of the undesirable results above referred to. This consists in passing a somewhat blunt pointed, curved and rather dull bistoury under the opened sheath of the tendon, from the first phalanx, whether it be finger or thumb, into the centre of the palm, and sweeping it gently round, so as to break down the inflamed synovial and cellular tissues, and furnish a free outlet for the fluids with which they are distended. Little force is given to the sweep of the bistoury, so that the tendons need not be cut. No arterial hæmorrhage will follow, for the swollen state of the hand separates the two arches—the radial arch being fixed by its perforating vessels, near the face of the metacarpal bones, and the ulnar being pushed off with the palmar fascia, laying as it does in front of the superficial flexor tendons. This I shall do, if by to-morrow I do not find the state of the palm much improved. For the present, I shall direct the hand and arm to be kept constantly sopped with a strong solution of the extract of lead and laudanum in water, and direct the patient to take the following mixture:

| | | |
|---|---|---|
| ℞. | Aqua Camphor. | |
| | Sp. Mindereri ana, | f.℥iij. |
| | Sp. Nitri. Dulcis, | f.ʒss. |
| | Morph. Acetat. | |
| | Ant. Tart. ana, | gr. i. |
| | Sac. Alb., | ʒj. |

In table-spoonful doses, every hour, for the purpose of quieting nervous irritation, giving the patient sleep, and reducing the fever by exciting free perspiration. If perspiration does not follow after the third dose, I would slightly increase the proportion of tart. antimony, to ward off febrile excitement. The patient, for the present, to be kept on a diet of toast and tea. The back of the same fingers, as well as the hand, is, as you see here, much swollen. This is but an extension of the disease from the palmar surface. Matter may form here, so as to render it necessary to make incisions for its exit. If it be necessary to do do this, in the back of the hand, I sometimes purposely include in the cut one of the superficial veins, in order to unload the vessels. As the disease extends round to the back of the hand, so will it sometimes invade the joints of the fingers, followed, occasionally, either by anchylosis, or the necessity of amputating the part. If the accident occur in a subject with a strong tendency to scrofula or phthisis, we may have fungous degeneration, like the white swellings of the larger joints, for we have here the same tissues to be involved. Even when neither of these cachexia are present, we sometimes have a spongy, fungous state of the tendinous sheaths, that we have to correct by touching lightly with caustic potash.

Still, under the treatment, above suggested, and which I have found, of all others, the most reliable in these cases, I have no doubt the patient will do well. There may remain a permanent stiffness of one of the fingers, if there be great injury to the tendons where they pass each other, but I feel very confident, that not only the patient's life, but her hand will be saved.

*Cases* 3 *and* 4. *Strabismus, (convergens and divergens.)*—We next have presented two cases of Strabismus, one female and one male, both adults—and both cases of double squint. In the former we have the internal or common form of the affection, strabismus convergens. In the other, we have the external squint, or strabismus divergens; a variety which I believe does not occur more than four or five times in a hundred cases of this deformity. This, from its comparative rarity, is a much more unpleasing affection than the internal variety; both eyes look outward like those of the bullock, and hence we have the term bull-eyed, applied by the vulgar to persons thus afflicted. You have seen me already operate, many times, for strabismus, during the session, and are familiar with the mode which I practise. I shall proceed to correct the deformity in both these cases to-day, and will take this occasion to enter briefly, though somewhat more fully than I have hitherto done, into the principles involved, and the rules by which I am guided in the operation. I do this the more willingly, because I think they are not so well understood generally as they should be, and because, from the imperfect success of the operation in the hands of many surgeons, there is a sort of general opinion in the profession out of the large cities, that the operation for strabismus has, in a great measure, been abandoned. Now, there is nothing more untrue than this.

I was among the earliest in the country to operate for this deformity, after the news of Dieffenbach's successes, in 1839, had reached us. I have continued to operate from that time to the present, and have certainly, in all, performed something like a thousand operations, and in patients of almost all ages between 5 and 60; and my most deliberate conviction is, that there is no operation in surgery that yields (when rightly managed) more gratifying results. It is one of the very few in which I feel inclined to promise a perfect cure, provided the patient will yield obedience to my after treatment. Too many are apt to believe that the whole of the operation consists merely in cutting a tendon. That any one can do ; but it is a small part of what we have to attend to. The character of the operation has to vary with nearly every case.

Much more time would be required than we can possibly spare to-day, as there are some other patients to be attended to, were I to undertake to cover the whole ground of necessary instruction. I will do the best I can by laying down some rules.

We must first start with a proper knowledge of the cause of the deformity. This consists in a disproportionate unrelaxing or spastic contraction of one of the straight muscles of the eyeball, when compared with its antagonist on the other side of the ball. This may be, and very commonly is in the first instance, confined to one eye, but as it destroys the parallelism in the axis of vision of the two organs, the other eye is sooner or later, and to more or less extent, made, sympathetically, to assume the same kind of distortion. The exact cause of the too forcible or unyielding contraction of one muscle is various. It seems to arise, occasionally, from the mere habit of imitation; by too exclusively keeping the eye fixed on near objects; from the forceful pressure of the finger against one ball, for the amusement of making objects look double; from the growth of a tumor, which prevents the eye from turning properly; and from a variety of like varying causes. But by far the most common of all, is the disturbance of the centric extremities of the nerves of the straight muscles by some congestion or inflammation of the meninges or substance of the brain, which so often occurs in scarlatina, measles, hooping cough, or cerebral fever. The result of this disturbance as the cause of squint, has been long known to the profession, but it was taught, and even so lately as in the last surgical lectures of Sir C. Bell, to be due to the paralysis of one muscle, and not to the over stimulation of its antagonist. This error in pathology fettered the hand of the surgeon, for over a paralytic muscle he had little means of control. Stromeyer and Dieffenbach showed as the general rule, the incorrectness of the opinion.

I see cases occasionally, in which the form of strabismus, (the divergent) as shown in this case, appears to be produced by a congestion of the cavernous sinus, through which runs, as you know, the nerves to the muscles of the eyeball, and with the internal wall of which the sixth pair of nerves is intimately connected. I have been several times consulted by patients, so far as I recollect all adults, for relief from a double vision, steadily increasing and of recent occurrence. In all these cases the squint was external, and was accompanied with a sort of amblyopic dulness of vision, undefined pain in the region of the head, increased intolerably by exposure to our summer sun, with more or less constipation and irregularity of the bowels. I have not, of course, any positive proof of the correctness of the pathology, but I have thought that this state of things was dependent upon a congestion of the choroid veins of the eye, and the cavernous sinus, accompanied by a sluggish circulation in the portal venous system, with which the German ophthalmologists teach that the choroid veins so directly sympathize.

Be that as it may, I have always been able to relieve these cases when recent, (and last winter I had an opportunity of showing the fact in this clinic,) by repeated alternate cupping and blistering to the back of the neck and temple, occasional thorough purging with blue mass and comp. ext. of colocynth, and giving daily douches of cold water to the head while the patient was resting up to the knees in hot water. If this affection, however, is not seen in its early state, I believe it is not possible to afford relief, except by dividing the tendon of the muscle, (the external rectus) which has undergone the spastic or unrelaxing contraction.

I will now in this case of internal squint, make the preparatory investigation necessary to the operation.

I first wish to determine if this is double, and if so, if the distortion is equal in both eyes. To do this accurately is difficult, for the patient can direct either eye at will straight towards an object, and the whole obliquity of both eyes will seem accumulated in one. The mode to which I resort, to determine this, in all forms of squint, is the following: I place myself in front of the patient, and direct him to roll the eyes as far as possible, alternately to the right and left. Now a person at middle age, with healthy eyes, can hide the white completely at the outer canthus and slightly bury the edge of the cornea at the inner. A young subject in which the tissues are always more yielding, can do more than this. If I find after repeated trials of this sort, that the movements of one eye are normal, and that in the other the cornea is buried too deeply in the inner canthus, and that a strip of the adnata, a sixth or an eighth of an inch, is left uncovered at the outer, then, I say the internal rectus of this eye is alone at fault, and its tendon must be divided.

We will also frequently observe in the faulty muscle, an involuntary and rapid twitching when the eye is turned strongly in the opposite direction. If, at the same time, I find that the eye at fault pretty generally rolls upwards and inwards, or downwards and inwards, I say, that a portion of the upper or lower rectus muscle is likewise concerned in the deformity, and may, after the operation on the internal rectus, require to have its tendon cut, and I locate my incision through the conjunctiva to suit.

I next observe carefully when the patient is making no unusual effort with the eyes, whether the lids open equally wide, or, in other words, if there is not a larger disk covered at the upper part of the cornea in one eye than the other. If so, I am sure that the eye that has the cornea most covered, is the one which has the shortened muscle and the squint, for the effect of the strabismic contraction is to sink the ball and leave the lids less widely separated. By applying these tests, we may find both eyes equally affected with strabismus, or affected in different degrees, and we must plan our operations accordingly. Here comes into requisition the judgment and skill of the operator, for the nice point to determine is, which tendon, and how far, he is to cut—for a wrong incision often leaves a deformity which it is hard to correct. By a close attention to these rules, one surgeon will be almost invariably successful, and another of equal, or even greater merits in other respects, will without it be occasionally very unfortunate.

If it be a clearly marked case of single squint, the course is plain; you have but the one eye to act upon, and you divide the conjunctiva over the insertion of the tendon, raise the tendon with the blunt hook and divide it neatly with the scissors near the sclerotic coat, so as to leave no protruding stump to the tendon. If the squint has been at the same time a little upwards or downwards, you must dilate the intermuscular fascia by a section in one of those directions. If after this the eye does not come straight, you should, provided it remains a little sunken, divide a portion of the superior or inferior rectus. But if the eye, without becoming straight, is a little more or equally prominent with the other, withhold the division of these muscles, for if you cut them you weaken the stays of the ball, and you will have the eye, though it may be straight, probably prominent and protruding—a result which has been too frequently produced by an attempt to cure a double squint, in the mistaken belief that one eye only was at fault, and which is the main cause that the operation has fallen into discredit with those who have witnessed such mishaps. You have now a squint in the other eye, and to that you must turn your attention. It cannot be very great, or you would have discovered it by the preparatory tests to which I have alluded. You have therefore to divide the corresponding tendon in the opposite eye, and this may be done in the *adult*, at the same sitting. It must, however, be done with caution. You have to cut the tendon of the internal rectus completely, or you gain nothing, for if a few fibres be left it will keep up its disturbing action on the ball. But you should not cut any portion of the intermuscular fascia, and ought to weaken as little as possible, the retaining force of the conjunctiva. Your object is to straighten the direction of this ball, without causing it sensibly to protrude.

For this purpose the sub-conjunctival operation of Guèrin, is the most appropriate, in which we make a puncture, and divide the tendon with this crooked knife beneath the surface, as in the subcutaneous process upon the tendons about the joints. Or wanting this instrument, it answers nearly as well, to make a horizontal incision of the conjunctiva, raise the tendon between the lips, and divide it with the knife or scissors under the edge of the membrane.

I have here stated one of the ordinary difficulties that occur in the operation, and the mode in which you may effect a perfect cure without causing in either eye a prominence beyond the other. We will very frequently have a double squint, so strongly and clearly expressed, that it will be best for us to act at once on each eye with the common vertical cut of the conjunctiva, limiting ourselves at first, however, to the division of the single tendon merely on each eye. After this is done, we then apply the preparatory tests above noticed, and see which eye requires, in order to bring both parallel and with an equal degree of prominence, to have the intermuscular fascia dilated upwards or downwards, or one of the adjoining straight muscles either partially or in extreme cases entirely cut. These incisions have frequently to be made upon both eyes, especially in cases of long standing, but not usually to an equal extent.

I have spoken of the operation as yet in reference to adults, and in cases of internal squint. The same remarks will apply to the operation for external strabismus, which is easier of performance, as the tendon on that side is uncovered for a longer space, and we have no plica semilunaris as at the inner canthus, into which or even immediately adjoining which, we are never to cut. Notwithstanding this, the cure of the external squint does not appear to me to be quite so easily, or so readily made, as that of the internal, and the cause of this appears to me to have been in some cases owing to an attendant paralytic weakness of the internal rectus muscle.

If you are treating young subjects, (and under 10 years of age I would not advise you to undertake the operation, though there may be very properly exceptions made to this rule,) you must be much more guarded in respect to the extent of your sections. For in young persons, the overstretched muscles have much greater recuperative energy, and this gradual increase of their power, seems extended over several days. The evil to be feared then is, that the muscle antagonising the one cut, will pull the ball so far over as to let the divided tendon become reattached too far back on the globe. There are, therefore, few cases in persons under puberty even of double squint, in which I should deem it judicious to operate on both eyes at one sitting. I would advise you to cut the tendon, and that merely on the eye most distorted, at the first sitting, and wait for for a few days or a fortnight, or even months in some cases, in order to ascertain fully what effect will be produced. It will then be found sometimes necessary to divide the tendon of the contracted muscle, on the opposite side. But in children you must always remember to make allowance for this recuperative force of the muscles, and not seek to make the eyes immediately straight. The proper degree of allowance to make, is one of the difficulties of this operation, which has to be graded by the judgment of the operator, according to the age of the patient, and the degree of the deformity.

It is true, in case an external squint should follow an operation for one of the opposite kind, you have it in your power to correct it by a subsequent division of the external rectus tendons, yet it is a most unpleasant result, which you ought, and may most usually avoid. One mode to which, when I particularly fear such a result, I have recourse, is, to stop considerably short of making the eyes straight, to reintroduce subsequently my blunt hook so as to loosen the reattaching tendon and nick the fascia above or below very cautiously with the scissors without enlarging the wound in the conjunctiva, until the balls begin to assume their proper positions.

In persons at, or past the middle period of life, we need have no fear of such a result, and the eyes should be made fully straight at the first sitting. I have operated in one case of unusual interest—a Mr. Samuel Solomons, near 60 years old, a patient of Dr. Mœhring's of this city. This man was partially amaurotic, and had so extreme an internal squint, that both corneæ were held hidden, save a small disk at the outer margin, in the internal canthi. The sight in one eye being a little better than the other, I selected it for an operation. By grasping the conjunctiva (which was red as scarlet, from its being always exposed) at the outer border of the cornea, and making traction upon it with the forceps, so as to be able to get a hook upon the inner side of the cornea, I managed to divide the internal rectus, the superior rectus and a portion of the inferior, with the intervening fascia, so as to get the cornea nearly round to the front of the orbit. By this means I was enabled to render the sight which had before been nearly useless, in a considerable degree serviceable to the patient. I mention this fact, as a striking illustration of the difference in density and resistance between the same structures at the opposite extremes of life. I have met with cases, however, even in young subjects, where the fascia and cellular structure at the inner canthus have been hardened by chronic inflammation, as when a wound has been inflicted or a carious bone detached, that rendered necessary a free dissection of the ball on one side, before the eye could be pulled straight by the antagonising muscles. But these are exceptional cases.

I have not in these remarks, spoken of any operation upon the oblique muscles, which we were at one time directed by many writers to cut in strabismus. The division of these muscles, is, at least in all ordinary cases, unnecessary, as I believe they are never directly concerned in the production of strabismus. I have several times divided them, in my early experience with this operation, without the slightest change in the ordinary movements of the ball. In two instances only was the division of the tendon of the superior oblique, attended with manifest advantage. In those cases, after having cut the internal rectus tendon, and dilated the fascia above and below, I found the ball held by some cellular tissue, in the direction of the superior oblique tendon. I drew this tendon out from the wound made through the conjunctiva, and divided it. It separated with a snap, and the hardened bands of cellular tissue, which had been held tense by it, were at once released, and the eye took its proper position in the orbit.

The operations were then performed on both patients in the manner above described, and with the usual successful result.

Two other operations were then practised, one for oscillation of the eye ball, and one for the radical cure of varicocele, but for want of time Prof. P. has deferred his explanatory remarks till another clinical occasion.

